# Oral Probiotic VSL#3 Prevents Autoimmune Diabetes by Modulating Microbiota and Promoting Indoleamine 2,3-Dioxygenase-Enriched Tolerogenic Intestinal Environment

**DOI:** 10.1155/2016/7569431

**Published:** 2015-12-08

**Authors:** Jayashree Dolpady, Chiara Sorini, Caterina Di Pietro, Ilaria Cosorich, Roberto Ferrarese, Diego Saita, Massimo Clementi, Filippo Canducci, Marika Falcone

**Affiliations:** ^1^Experimental Diabetes Unit, Diabetes Research Institute, Division of Immunology, Transplantation and Infectious Diseases, IRCCS San Raffaele Scientific Institute, Via Olgettina 60, 20132 Milan, Italy; ^2^Microbiology Unit, IRCCS San Raffaele Scientific Institute, Via Olgettina 60, 20132 Milan, Italy; ^3^Department of Biotechnology and Life Sciences, University of Insubria, 21100 Varese, Italy

## Abstract

The gut microbiota modulates the autoimmune pathogenesis of type 1 diabetes (T1D) via mechanisms that remain largely unknown. The inflammasome components are innate immune sensors that are highly influenced by the gut environment and play pivotal roles in maintaining intestinal immune homeostasis. In this study we show that modifications of the gut microbiota induced by oral treatment with Lactobacillaceae-enriched probiotic VSL#3, alone or in combination with retinoic acid (RA), protect NOD mice from T1D by affecting inflammasome at the intestinal level. In particular, we show that VSL#3 treatment inhibits IL-1*β* expression while enhancing release of protolerogenic components of the inflammasome, such as indoleamine 2,3-dioxygenase (IDO) and IL-33. Those modifications of the intestinal microenvironment in VSL#3-treated NOD mice modulate gut immunity by promoting differentiation of tolerogenic CD103^+^ DCs and reducing differentiation/expansion of Th1 and Th17 cells in the intestinal mucosa and at the sites of autoimmunity, that is, within the pancreatic lymph nodes (PLN) of VSL#3-treated NOD mice. Our data provide a link between dietary factors, microbiota composition, intestinal inflammation, and immune homeostasis in autoimmune diabetes and could pave the way for new therapeutic approaches aimed at changing the intestinal microenvironment with probiotics to counterregulate autoimmunity and prevent T1D.

## 1. Introduction

The human intestinal microbial ecosystem includes at least 1,000 distinct bacterial species that possess 100-fold more genes compared with their human hosts [1]. This complex microbial system modulates the pathogenesis of different diseases, including allergies, tumors, and autoimmune diseases such as T1D with either beneficial or detrimental effects [2]. In humans, T1D susceptibility has been linked to compositional changes in the gut microbiota and, specifically, with a significant increased representation of bacteria of the Bacteroidetes phylum and a decrease in the number of* Bifidobacterium*,* Lactobacillus*, and* Clostridium* strains [3–5]. Studies in preclinical models of T1D have indicated that the gut microbiota plays a key role in controlling disease onset and severity. For example, the absence of MyD88, an adaptor molecule involved in TLR signaling, protects NOD mice from autoimmune T1D by inducing a protective microbiota profile characterized by a low Firmicutes/Bacteroidetes ratio and Lactobacillaceae strain enrichment [6]. The beneficial effect of Lactobacillaceae strains in T1D was also proven by the observation that treatment of NOD mice with VSL#3, a probiotic enriched in Lactobacillaceae largely used to modulate microbiota composition in humans, prevents T1D [7]. The mechanisms underlying the influence of the microbiota on T1D pathogenesis have not yet been clarified. Recent evidence indicates that T cells originating in the gut mucosa can modulate autoimmunity at sites distal from the intestines such as the central nervous system (CNS) in experimental allergic encephalomyelitis (EAE), the experimental model of multiple sclerosis [8]. Similarly, gut microbiota modifications can affect autoimmune T1D by altering T cell immunity within the intestinal mucosa and in the pancreatic lymph nodes (PLN) and islets [9]. In support of this hypothesis, there are some lines of evidence showing that gut immunity is altered in T1D. For example, alterations in immune homeostasis and signs of immune dysregulation such as increased expression of MHC II and inflammatory cytokines have been found in the intestines of both T1D patients and preclinical T1D models even before clinical onset of the disease [10–16]. In addition, intestinal immune regulation, specifically differentiation of FoxP3^+^ Treg cells in the intestinal mucosa, is significantly impaired in T1D patients [17]. These observations have led to the hypothesis that intestinal immunity may be important for regulation of T1D. That was also confirmed from finding that a large fraction of the T cells infiltrating the pancreatic islets of T1D patients and NOD mice originate in the intestine and express gut-homing receptors, such as CCR9 and *α*4*β*7 [18, 19]. Thus, the microbiota composition may modulate T1D by altering intestinal immunity and immune regulation. Proinflammatory microbial species can elicit specific immune responses that trigger inflammation and promote T1D by destroying gut immune homeostasis. On the contrary, beneficial microbial species can confer protection against T1D by inducing immune tolerance in the gut mucosa and a protective Teff/Treg cell ratio in the intestine, and in distal organs, such as the pancreatic islets.

Dietary factors, like vitamins, can similarly affect T1D pathogenesis by modulating gut immunity. A vitamin A-rich diet as well as the administration of vitamin A and its metabolite, all-*trans*-retinoic acid (RA), has been shown to prevent autoimmune T1D in preclinical models [20, 21]. Vitamin A and its metabolite RA are crucial for maintenance of immune tolerance in the intestine; thus their administration may counterregulate autoimmune T1D by regulating the reciprocal relationship between Treg and Teff cells in the intestine.

Here, we demonstrate that administration of the Lactobacillaceae-enriched VSL#3 probiotic, alone or in combination with RA, prevents T1D in NOD mice by enriching the local microbiota with Lactobacillaceae strains and by inducing substantial modifications in the microbiota composition with increase in Clostridia and Rikenellaceae species and reduction in the Bacteroidetes strain S24-7. In addition, VSL#3 administration and the resulting microbiota modifications generate a protolerogenic intestinal microenvironment with high expression of IDO and low expression of inflammatory IL-1*β*. The VSL#3-induced protolerogenic microenvironment promoted CD103^+^ DC differentiation and reduced the Teff/Treg cell ratios within the gut mucosa, mesenteric lymph nodes (MLN), and PLN, thereby modulating T1D pathogenesis. 

## 2. Materials and Methods

### 2.1. Mice

Four-week-old NOD mice were purchased from Charles River Laboratories (Calco, Italy) and maintained under specific pathogen-free conditions in the animal facility of San Raffaele Scientific Institute. All animal experiments were performed with permission from the Institutional Animal Care and Use Committee (IACUC) according to the rules of the Italian Ministry of Health.

### 2.2. Treatments

VSL#3 (VSL Pharmaceuticals, Ft. Lauderdale, FL, USA) is a probiotic compound containing 3 × 10^11^/g of viable lyophilized bacteria, including Bifidobacteriaceae (*B. longum*,* B. infantis*, and* B. breve*), Lactobacillaceae (*L. acidophilus, L. paracasei*,* L. delbrueckii *subsp.* Bulgaricus*, and* L. plantarum*), and* Streptococcus thermophilus*. VSL#3 was administered through oral gavage three times a week starting at 4 weeks until 20 weeks of age. A group of VSL#3-treated NOD mice also received 50 *μ*g of all-*trans*-RA (Sigma-Aldrich, St. Louis, MO, USA) twice a week by intraperitoneal injection.

### 2.3. Microbiota Profiling

A microbial survey of the fecal community members was performed by pyrosequencing of barcoded 16S rRNA gene amplicons. DNA was isolated from feces collected at 12–14 weeks of age by using a QIAamp DNA Stool mini kit (Qiagen, Germantown, MD, USA). Microbiome characterization of the stool samples was performed by amplification of the V3-V3 region of 16S rRNA using universal primer pairs. The gut metagenome was analyzed using QIIME. Sequences shorter than 250 bp were not analyzed. Chimeric sequences were excluded using ChimeraSlayer software, and taxonomies were assigned within QIIME using RDP.

### 2.4. Inflammasome Analysis by Quantitative Real-Time PCR (q-RT-PCR)

Total RNA was isolated from intestinal tissue with an RNeasy Mini Kit (Qiagen, Germantown, MD, USA). Total RNA was reverse transcribed, and qPCR was performed with an iQ5 Real-Time Detection System (Bio-Rad, Hercules, CA, USA) using specific primers [22]. The values were expressed as relative fold differences and were normalized against the* Gapdh *housekeeping gene according to the 2-ΔΔCT method [23].

### 2.5. Cell Isolation

Mononuclear cells were isolated from the SI-Lp and C-Lp as previously described [24, 25]. In more detail, after the removal of Peyer's patches, small intestines and colons were flushed with PBS, opened longitudinally, and predigested twice with 5 mM EDTA and 1 mM DTT for 20 min at 37°C. After removing epithelial cells and fat tissue, the intestines were cut into small pieces and incubated in HBSS containing 0.5 mg/mL Collagenase D (Roche), 1 mg/mL Dispase II (Roche), and 5 U/mL DNase I (Sigma) for 20 min at 37°C in a shaking incubator. The digested tissues were washed, resuspended in 5 mL of 40% Percoll (Sigma), and overlaid on 2.5 mL of 80% Percoll in a 15 mL Falcon tube. Percoll gradient separation was performed by centrifugation at 1000 g for 20 min at 20°C. The interface cells were collected and used as Lp lymphocytes. Splenocytes and lymph node cells were isolated by mechanical disruption of the tissue. An additional digestion step with 1 mg/mL Collagenase IV (Gibco) for 20 min at 37°C was performed to increase dendritic cell extraction. Pancreatic intraislet lymphocytes were isolated by 3-step digestion with 1 mg/mL Collagenase IV (Gibco) followed by extensive washing with HBSS containing 5% FBS.

### 2.6. Flow Cytometry Analysis

Intracellular staining and FACS analysis of the single cell suspensions were performed to analyze the cytokine secretion profile of the T helper cells. Briefly, single cell suspensions were stimulated with 50 ng/mL phorbol 12-myristate 13-acetate (PMA) and 1 *μ*g/mL ionomycin (both from Sigma-Aldrich, St. Louis, MO, USA) for 4 hours in the presence of 10 *μ*g/mL Brefeldin A (Sandoz, NJ, USA) followed by fixation and permeabilization with 2% Paraformaldehyde (PFA) and 0.5% Saponin (both from Sigma-Aldrich, St. Louis, MO, USA). After stimulation, the cells were stained with a PerCP-Cy5.5 conjugated anti-CD4 mAb, fixed and permeabilized (Cytofix/Cytoperm kit, BD Biosciences, San Jose, CA, USA), and stained with FITC-conjugated anti-IFN-*γ* and AlexaFluor647-conjugated anti-IL17A mAbs. To detect the FOXP3 intracellular marker, cells were stained with PerCP-Cy5.5 anti-mouse CD4 and APC-conjugated anti-mouse CD25 mAbs against surface molecules and then fixed (Foxp3 Staining Buffer set, eBioscience, San Diego, CA, USA), permeabilized, and stained with an anti-FOXP3 mAb. To assess the relative percentages of the different intestinal DC subsets, cells were stained with a V450-conjugated hamster mAb anti-mouse CD11c, a PE-Cy7 conjugated rat mAb anti-mouse CD11b, and a PE-conjugated rat mAb anti-mouse CD103 (all from BD Biosciences, San Jose, CA, USA). For CX3CR1 staining, we used a primary rabbit polyclonal antibody, anti-CX3CR1 (Abcam, Cambridge, UK), and a secondary FITC-conjugated F(ab′)2 anti-rabbit IgG antibody (eBioscience, San Diego, CA, USA). The flow cytometry data were acquired using a FACScan and analyzed with CellQuest software (BD Biosciences, CA, USA).

### 2.7. Cytokine Secretion

To analyze the cytokine secretion profile of the intestinal DCs, CD11c^+^ cells were isolated by magnetic separation from intestinal single cell suspensions and stimulated* in vitro* with 1 *μ*g/mL LPS for 20 hrs. IL-1*β*, IL-12, and IL-10 in the cell culture supernatants were quantitated with a BD Cytometric Bead Array (CBA from BD Biosciences, CA, USA) and FACS analysis following the manufacturer's protocol. The data were analyzed with FCAPArray software v1.0.1 (Soft Flow, St. Louis Park, MN, USA).

### 2.8. Diabetes Protection

Diabetes was monitored by testing blood glucose levels with a GB35 Ascensia Breeze 2 glucometer (Bayer, Pittsburgh, PA, USA). To assess histopathological signs of diabetes, pancreata were fixed in 10% formalin, embedded in paraffin, and sectioned into 4 *μ*m slices. The pancreata sections were then stained with hematoxylin-eosin and scored for insulitis.

### 2.9. Statistical Analysis

Statistical analysis of the microbiota profiling data was performed on the proportional representation of the taxa (summarized to phyla, class, order, family, and genus levels) using one-way ANOVA test with Bonferroni's correction. Statistical significances of the differences in the percentages of positive cells were calculated by application of unpaired 2-tailed Student's *t*-test using Graphpad Prism software. Statistical significance between survival curves was calculated by the log-rank (Mantel-Cox) test using Prism 5.0 software. *p* values of less than 0.05 were considered statistically significant.

## 3. Results

**Figure 1:**
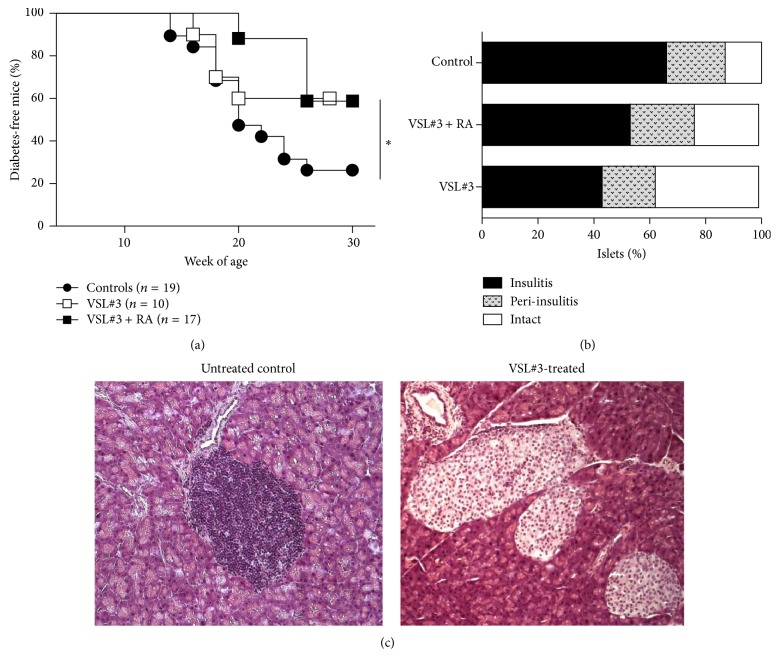
All-*trans*-retinoic acid (RA) does not enhance the protective effect of the VSL#3 probiotic against autoimmune T1D. (a) VSL#3 (14 mg/kg of body weight) was administered orally three times per week alone (*n* = 10) or in combination with RA (i.p.) (*n* = 17). A control group (*n* = 19) received sterile PBS by oral gavage. An additional group received RA alone i.p. All treatments were initiated at 4 weeks of age. Diabetes incidence was evaluated by measuring blood glucose levels staring at 12 weeks of age. For all groups, diabetes was diagnosed after two consecutive measurements of glycemia ≥ 250 mg/dL. Mice were pooled from two independent experiments. The log-rank (Mantel-Cox) test was used for the comparison of diabetes incidence rates between the different groups (^*∗*^ *p* value < 0.05). (b) The histopathological signs of diabetes were evaluated in pancreatic sections stained with hematoxylin-eosin. The islets were ranked as free of insulitis (intact islets), affected by peri-insulitis (mononuclear cells in the connective tissue around the islets), or affected by insulitis (mononuclear cell infiltration > 20% of the islet). (c) Two representative images of the histopathological analysis are shown.

**Figure 2:**
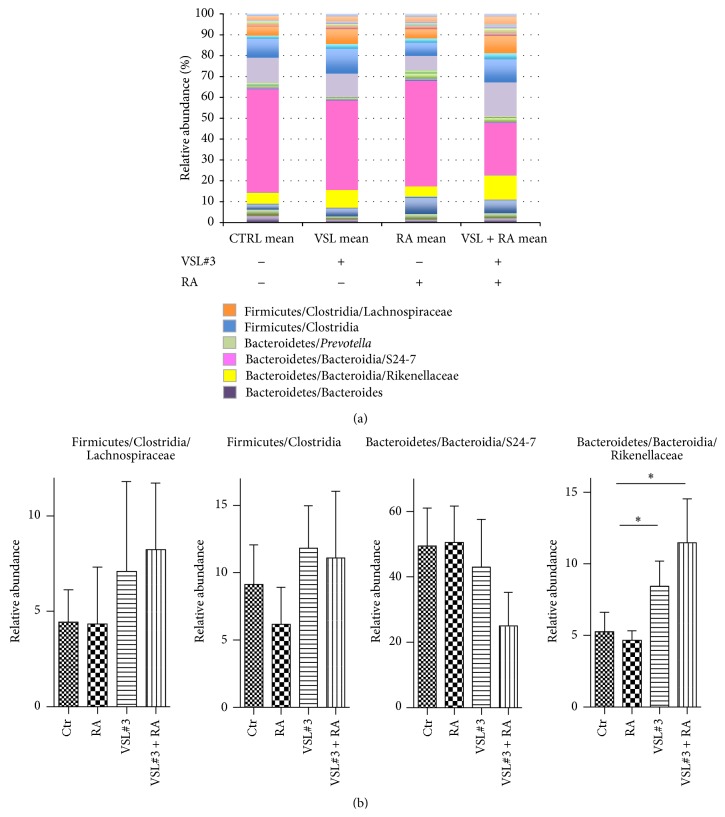
VSL#3 probiotic administration protects against T1D by changing the intestinal microbiota composition of NOD mice. Microbiota profiling by 16S metagenomic analysis was performed on microbiota samples obtained from female NOD mice treated with VSL#3 (VSL#3), RA alone (RA), or VSL#3 + RA or from untreated (Ctr) mice. Luminal contents from individual mice were collected at 12–14 weeks of age before onset of clinical diabetes (hyperglycemia), and a microbial survey of the metabolically active fecal community members was performed by pyrosequencing of the barcoded 16S rRNA gene. Between 15000 and 20000 sequences/samples were used in the analysis. Good coverage indices of >0,99 were observed for all samples. (a) Relative abundances of the different murine intestinal families in the four groups of mice. (b) The data are expressed as the mean ± SEM of the relative abundances of the major murine intestinal phyla. ^*∗*^ *p* < 0.05 by one-way ANOVA test with Bonferroni's correction.

A previous report has shown that administration of the probiotic VSL#3 confers protection against T1D in NOD mice [7]. We repeated the experiment to assess the mechanism underlying VSL#3-mediated protection and to verify whether it could be enhanced by simultaneous administration of all-*trans*-RA, a vitamin A metabolite that critically modulates gut immunity [26]. NOD mice received the VSL#3 probiotic via weekly gavage administration starting at 4 weeks of age until 20 weeks of age. An additional group of NOD mice received VSL#3 in combination with RA while the control group received PBS only by gavage. As shown in Figure 1(a), we confirmed that administration of VSL#3 alone significantly protected NOD mice from T1D (*p* < 0.05) and the combination therapy with VSL#3 and RA did not increase protection from autoimmune T1D. The protective effect of VSL#3 treatment was confirmed at the histological level and NOD mice receiving VSL#3 with or without RA showed reduced degree of insulitis compared to their nontreated littermates (Figures 1(b) and 1(c)). Next, we aimed to identify the mechanisms underlying VSL#3- and VSL#3 + RA-mediated T1D protection. Although it was hypothesized that VSL#3, which is a probiotic enriched in Lactobacillaceae strains, prevented T1D by favoring colonization of the intestine of NOD mice with beneficial bacteria, comparative analysis of the microbiota profiles in VSL#3-treated and untreated NOD mice has never been performed. We analyzed at the phylum, class, order, family, and genus level the composition of the intestinal fecal community by ultradeep pyrosequencing of barcoded 16S rRNA gene amplicons in NOD mice treated with VSL#3 or VSL#3 + RA or in control NOD mice that were either untreated or treated with RA alone. As shown in Figure 2, administration of VSL#3 to NOD mice with/without RA induced a substantial change in the microbiota profile by increasing the relative abundance of some bacterial strains such as Clostridia species, which have strong protolerogenic properties and the capacity to induce immune tolerance and to reduce inflammation within intestinal tissues [27, 28]. On the other hand, bacterial strains belonging to the Bacteroidaceae family (strain S24-7) were strongly reduced in the VSL#3-protected mice, thus suggesting that those strains may have proinflammatory effects and a pathogenic role in T1D. Other strains of Bacteroidetes, such as the Rikenellaceae strains, were increased in the VSL#3-protected mice, indicating that the beneficial and pathogenic microbiota profiles were not regulated at the family level but were possibly linked to the relative abundance of the specific bacterial strains.

**Figure 3:**
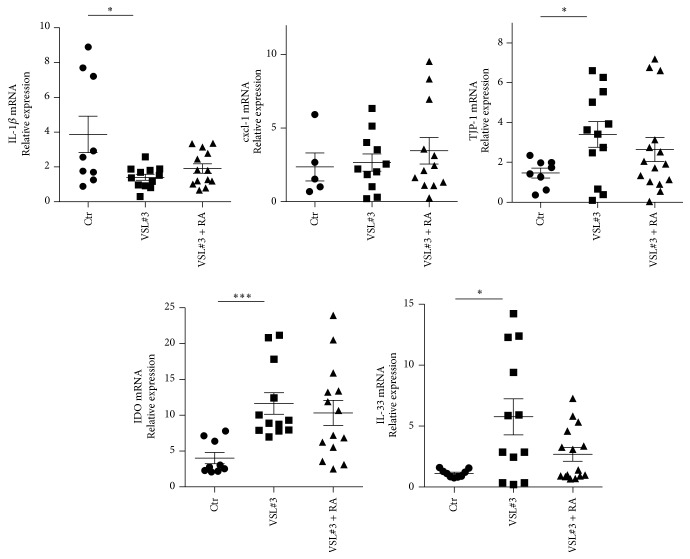
VSL#3 treatment halts intestinal inflammation and induces a protolerogenic intestinal environment. Intestinal mucosa tissues isolated from 12-week-old female NOD mice untreated (*n* = 9) or treated with VSL#3 alone (VSL#3; *n* = 12) or in combination with RA (VSL#3 + RA). mRNA expression levels of the indicated cytokines and inflammasome components were measured by quantitative real-time PCR. Values represent the relative fold differences of the individual mice normalized against expression of a housekeeping gene (*Gapdh*). ^*∗∗∗*^ *p* < 0.0005, ^*∗*^ *p* < 0.05 by Student's *t*-test.

Next, we assessed whether the microbiota modifications induced by VSL#3 and VSL#3 + RA treatments affected T1D by altering gut immune homeostasis and Treg/Teff cell balance in the intestinal mucosa, PLN, and pancreatic islets. To this aim, we evaluated the cytokine/chemokine profile and assessed the immune activation state within the intestinal mucosa of VSL#3-treated, VSL#3 + RA-treated, and untreated NOD mice (Figure 3). Our data show that the VSL#3 treatment induces a significant reduction in expression of cytokines associated with inflammasome activation, such as IL-1*β*. TJP-1 is a gene that codes for the tight junction protein zonula occludens 1 (ZO1), a crucial protein for maintaining the epithelial barrier integrity [29]. We found an overexpression of TJP-1 gene in VSL#3-treated NOD mice that may be an index of increased intestinal barrier integrity. At the same time, we detected a significant increase in expression of indoleamine 2,3-dioxygenase (IDO), an inflammasome component that, by regulating tryptophan catabolism, plays important immune modulatory functions in the intestine [30]. Also, IL-33, an alarmin that is also involved in tissue inflammation but also in epithelial regeneration and mucosal wound healing [31, 32], was increased in VSL#3-protected mice.

**Figure 4:**
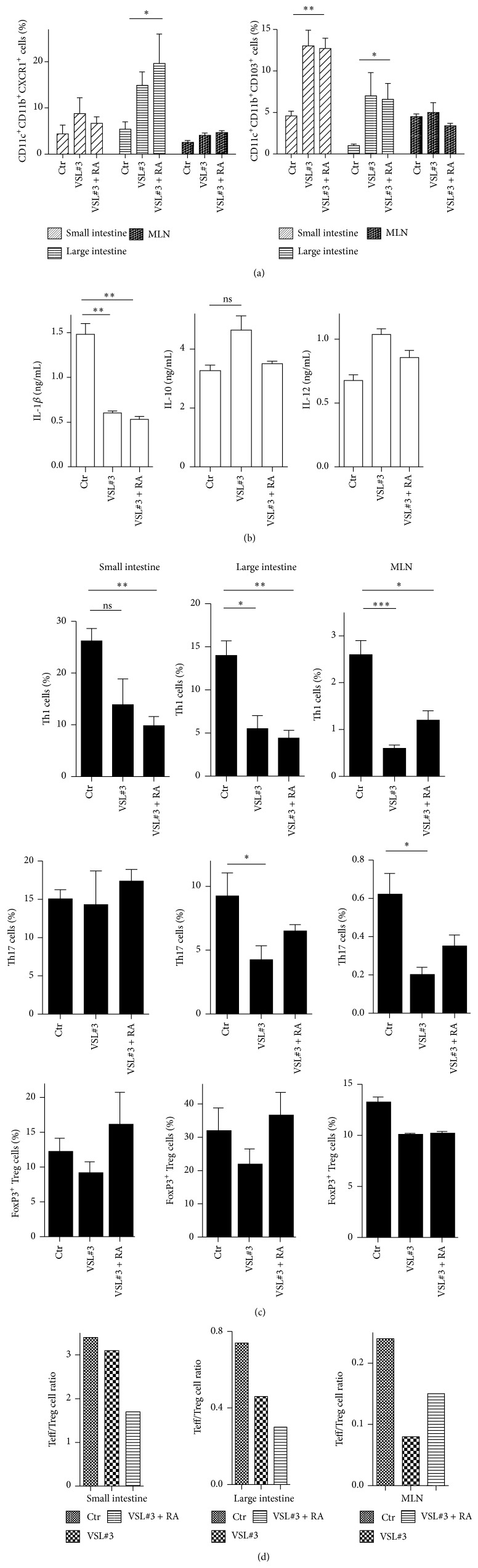
VSL#3 inhibits inflammation by inhibiting effector Th1/Th17 cell differentiation in the gut mucosa. (a) Percentages of different intestinal APC subsets in the small intestine (duodenum, jejunum, and ileum), large intestine (colon), and MLN (mesenteric lymph nodes) of 12-week-old NOD mice that were either untreated or treated with VSL#3 or VSL#3 + RA. Treatments were initiated at 4 weeks of age. (b) The cytokine secretion profile of the intestinal DCs from the gut mucosa of the three groups of NOD mice. CD11c^+^ DCs were purified by magnetic selection and stimulated for 20 hours with LPS (1 *μ*g/mL). Supernatants were collected and analyzed for cytokine content. The data are expressed as the mean ± SEM of triplicate experiments. (c) The percentages of Th1 cells, Th17 cells (CD4^+^CD3^+^IL-17A^+^), and FoxP3^+^ Treg cells (CD4^+^CD25^+^FoxP3^+^) were measured by intracellular staining and FACS analysis and expressed as the mean ± SEM. (d) The data are expressed as Teff (Th1 + Th17)/Treg cell ratios. ^*∗∗∗*^ *p* < 0.0005; ^*∗∗*^ *p* < 0.005; ^*∗*^ *p* < 0.05 by Student's *t*-test.

The inflammatory milieu that is present in the intestines of diabetic NOD can alter immune homeostasis and unbalance the Treg/Teff ratio at the local and systemic levels. Intestinal DCs are crucial to drive T cell differentiation towards an effector (Th1/Th17 cells) or regulatory (FoxP3^+^ Treg cells) phenotype, so VSL#3 may prevent T1D by reducing the inflammatory milieu and favoring differentiation of antigen-presenting cells (APCs) with tolerogenic function. To analyze the involvement of intestinal APCs in VSL#3-mediated protection from T1D, we analyzed the phenotypes of intestinal DCs obtained from VSL#3-treated, VSL#3 + RA-treated, and control NOD mice. Our FACS analysis revealed that the VSL#3-treated and VSL#3 + RA-treated NOD mice have higher percentages of protolerogenic DC subsets, such as CD11c^+^CD11b^+^CXCR1^+^ cells and CD11c^+^CD11b^+^CD103^+^ DCs in the intestinal mucosa and MLN compared to the untreated controls (Figure 4(a)). In addition, the intestinal DCs of the VSL#3-treated and VSL#3 + RA-treated NOD mice secreted lower amounts of proinflammatory cytokines, such as IL-1*β*, while release of protolerogenic cytokines, such as IL-10, was slightly but not significantly increased (Figure 4(b)). Next, we assessed whether modification of the DC subsets in the intestinal mucosa of VSL#3-treated NOD mice affected the Teff/Treg cell differentiation ratio. We analyzed the presence of effector T helper cells (Th1 and Th17 cells) and regulatory T cell subsets (FoxP3^+^ Treg cells) in the small and large intestines as well as in the MLN of the different groups of mice. Our analysis revealed a significant reduction in the percentage of effector Th1 cells in all of the intestinal tracts and MLN of the VSL#3-treated and VSL#3 + RA-treated animals in comparison with the untreated NOD controls (Figure 4(c)). Notably, the Th1 cell reduction was more significant in the VSL#3 + RA-treated versus the VSL#3-treated NOD mice. Additionally, while the VSL#3 treatment had a minimal effect on FoxP3^+^ Treg cell differentiation (Figure 4(c)), simultaneous administration of RA resulted in increased Treg cell differentiation and a more evident reduction in the Teff/Treg cell ratio in the intestinal mucosa of the VSL#3 + RA-treated NOD mice compared with their NOD littermates treated only with VSL#3 (Figure 4(d)). Overall, our data indicate that administration of VSL#3 alone or, more significantly, in association with RA restores gut immune homeostasis by modulating DC function and reducing the Teff/Treg cell ratio.

**Figure 5:**
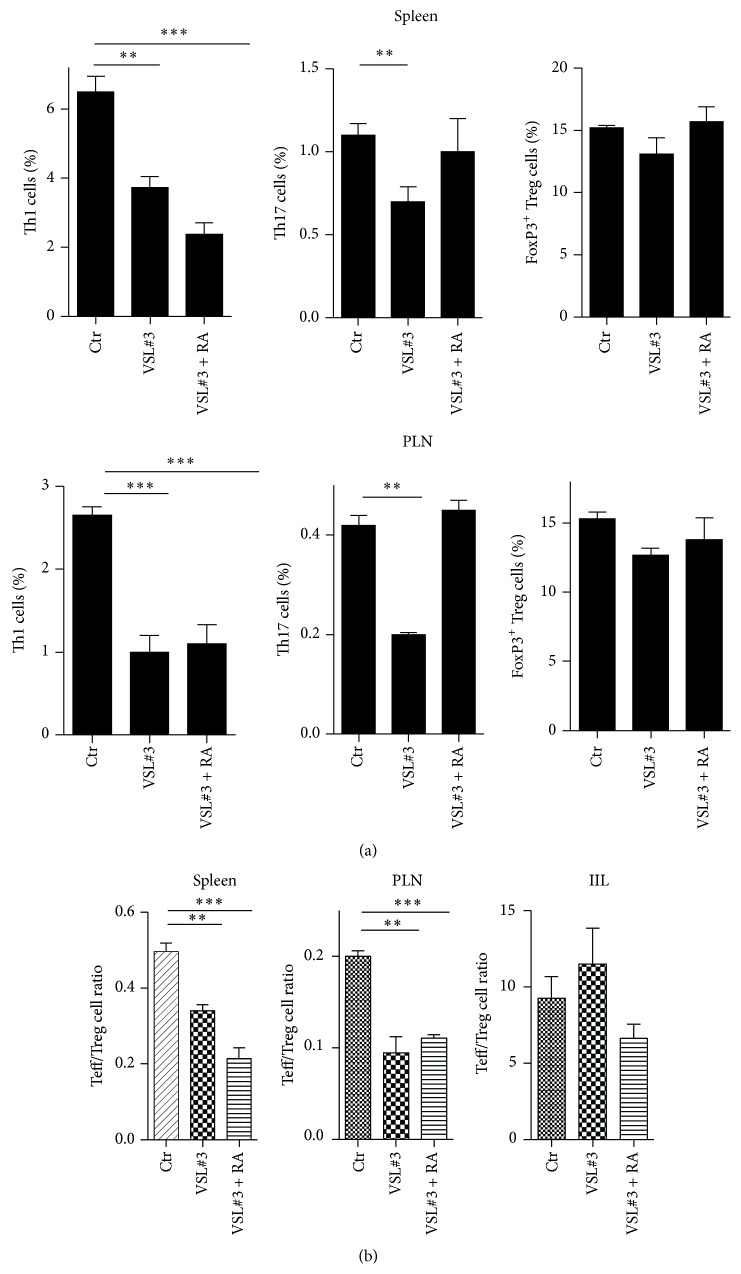
Restoration of immune homeostasis in the intestinal mucosa of VSL#3-treated NOD mice led to reduced Teff/Treg cell ratios at the systemic level as well as within the pancreatic lymph nodes (PLN) of NOD mice. (a) The percentages of different T helper cell subsets were measured in the total splenocytes (spleen) and PLN cells. Spleens and PLN were isolated from untreated (Ctr), VSL#3-treated (VSL#3), and VSL#3 + RA-treated NOD mice at 12 weeks of age (*n* = 4). The percentages of Th1, Th17, and FoxP3^+^ Treg cells were measured and expressed as the mean ± SEM. (b) The percentages of different T helper cell subsets were measured in the splenocytes (spleen), PLN cells, and islet infiltrating lymphocytes (IIL). The data are expressed as the Teff (Th1 + Th17)/Treg cell ratios. ^*∗∗∗*^ *p* < 0.0005, ^*∗∗*^ *p* < 0.005 by Student's *t*-test.

Self-reactive T cells that are found within pancreatic islet infiltrates in NOD mice and T1D patients have intestinal origins as demonstrated by their expression of gut-homing receptors such as *α*4*β*7 [18, 19]. Hence, we assessed whether the tolerogenic effect of VSL#3 and VSL#3 + RA administration on gut immunity can also modify the Treg/Teff cell balance at sites of autoimmunity, that is, the PLN and pancreatic islets of NOD mice.  Figure 5(a) shows that the percentages of effector Th1 cells and Th17 cells are reduced not only in the intestines but also at the systemic level in the spleen and PLN of the VSL#3-treated NOD mice (Figure 5(b)). Similar to what was observed in the intestines, the reduction in the Teff/Treg cell ratio was more significant when treatment with RA was associated with VSL#3 administration (Figure 5(b)). However, although the lymphocyte infiltrates and total number of T cells found in the pancreatic islets were lower in the VSL#3-treated and VSL#3 + RA-treated NOD mice, the Teff/Treg cell ratios were not significantly reduced in the pancreatic infiltrates of these mice (Figure 5(b)).

## 4. Discussion

Our data show that VSL#3 prevents T1D by modulating the gut environment both at the microbiota and immune cell levels. We demonstrate that VSL#3 and VSL#3 + RA administration to NOD mice not only enriches the gut microbiota of protective Lactobacillaceae species but provokes substantial modifications of the microbiota profile with enhancement of different protolerogenic species. For example, VSL#3-treated NOD mice showed an increased representation of Clostridia species that belong to the Firmicutes phylum and have an important protolerogenic effect by inducing FoxP3^+^ Treg cell differentiation in the intestinal mucosa [27, 28]. In VSL#3-treated NOD mice, we also found increased representation of other species that potentially provide protection against inflammation and autoimmunity, such as the Rikenellaceae species of the Bacteroidetes phylum. The VSL#3 treatment not only increased the presence of protolerogenic species but also reduced the relative abundances of species like Bacteroidetes strain S24-7 that is present in the microbiota of NOD mice and may be associated with gut inflammation and gut immunity alterations in those mice.

Although several studies have indicated that gut microbiota modifications can affect T1D pathogenesis, the mechanism underlying this modulatory effect remains unclear. In this study, we show that modifications of the microbiota profile induced by VSL#3 administration significantly reduced IL-1*β* expression in the intestinal mucosa of the NOD mice. IL-1*β* belongs to the inflammasome family, which is a group of protein complexes that are able to sense both microbial and damage-associated molecular patterns (DAMPs) and to initiate a potent innate, antimicrobial immune response [30]. The interaction of these components sustains the maintenance of the delicate equilibrium necessary to maintain intestinal immune homeostasis. IL-1*β*, which is secreted by lamina propria mononuclear cells (LPMCs) as well as tissue histiocytes and macrophages, is a strong marker of intestinal inflammation, and its tissue levels closely correlate with the degree of observed mucosal inflammation and necrosis in IBD patients [33]. Hence, we believe that expression of IL-1*β* in the intestinal mucosa of NOD mice is a clear marker of inflammation. This finding is in line with previous results showing that the intestinal mucosa of NOD mice possesses clear signs of immune activation and inflammation, such as increased expression of MHC II and inflammatory cytokines IL-1*α* and IFN-*γ* as well as lymphocyte infiltration and alterations in gut permeability [11–16]. Importantly, our data demonstrate that protection from T1D in VSL#3-treated NOD mice is associated with a significant decrease in IL-1*β* expression, thus suggesting a causative link between inflammasome activation, inflammation in the intestine, and autoimmune pathogenesis of T1D.

Together with decreased expression of IL-1*β*, we also found overexpression of different inflammasome components such as IDO and IL-33 in the intestinal mucosa of VSL#3-treated NOD mice. These inflammasomes, which are also released upon activation of innate sensors of danger, not only do contribute to amplification of the inflammatory process but are also crucial to maintain immune homeostasis at the intestinal level, restraining excess inflammation as well as favoring tissue regeneration [30]. IDO is an enzyme that mediates immune regulation via local metabolic changes in the immediate microenvironment and local tissue milieu [34]. Specifically, IDO suppresses T cell immunity by limiting the local tryptophan concentration, thus reducing effector Th1 and Th17 cell differentiation (direct effect) and also generating tryptophan metabolites that trigger* de novo* differentiation of regulatory T cells (indirect effect) [35]. The choice between immunity and tolerance in the intestine depends upon local factors that affect the balance between the immunosuppressive actions of the IDO pathway and local proinflammatory signals. In our system, VSL#3 administration, by altering the microbiota composition, shifts the balance toward IDO expression and tolerance induction. IDO overexpression in the VSL#3-treated NOD mice seems to act mostly through a direct effect that results in a significant reduction in the effector Th1 and Th17 cells. The indirect IDO modulation of regulatory FoxP3^+^ T cells is less evident in our system since we did not detect significant differences in FoxP3^+^ Treg cell differentiation in the gut mucosa of VSL#3-treated versus untreated NOD mice. We also detected a significant increase in the expression of IL-33 in the intestinal tissues of the VSL#3-treated NOD mice. IL-33 is an inflammasome component that plays a dichotomous role in the intestines, possessing either a proinflammatory or protective tissue regenerating function [31, 32]. In our model, IL-33 may also act through favoring Th2 cell bias [36] and reducing Th1 cell differentiation, thus cooperating with IDO in limiting effector Th1 cell differentiation in the intestines of VSL#3-treated NOD mice.

Our analysis also revealed increased expression of TJP-1 in the intestinal mucosa of VSL#3-treated NOD mice. This protein, which is also called zonulin-1 (ZO-1), plays an essential role in organizing the intestinal tight junction [29], and its defect has been linked to altered gut permeability in preclinical models of T1D [37]. Because previous reports have associated loss of intestinal barrier function to autoimmunity in T1D [38, 39], our data suggests that VSL#3 may counterregulate T1D not only by dampening inflammation but also by playing a direct beneficial role in the integrity of the intestinal tight junction barrier in VSL#3-treated NOD mice.

The effect on the inflammatory environment of the VSL#3 probiotic can be integrally related to modulation of intestinal DCs. Intestinal DCs are instrumental to sense environmental modifications in the gut mucosa and acquire a specific inflammatory or tolerogenic functional phenotype to build adaptive immune response against pathogens or induce immune tolerance against innocuous microbiota and food components. For example, intestinal DCs that express the surface marker CD103 and have tolerogenic function through secretion of soluble factors such as IDO and IL-10 [40, 41]. On the other hand, CD11c^+^CX3CR1^+^ cells are involved in transfer of the soluble luminal antigens to CD11c^+^CD103^+^ DCs via the gap junctions to induce immune tolerance [42]. Importantly, we found that the microbiota modifications induced by VSL#3 in the NOD mice reduced the increased inflammatory environment and IL-1*β* by modulating DC functional phenotype and increasing the percentages of tolerogenic CD11c^+^CD11b^+^CX3CR1^+^ and CD11c^+^CD11b^+^CD103^+^ cells.

Previous studies have shown that intestinal inflammation and microbiota alterations can be linked to T1D pathogenesis but the mechanisms underlying this modulation are still unclear. Our data indicate that gut environmental modifications in VSL#3-treated NOD mice directly affect T cell subsets and, specifically, the Teff/Treg cell ratio within the intestinal mucosa but also within the PLN, the site where the autoimmune response is regulated in T1D. A large body of evidence supports the notion that T cell subsets that originate in the gut travel through the peripheral lymphoid system and reach distal tissues, where they modulate immune-mediated diseases and autoimmunity [43] and a direct immunological axis between the gut mucosa and PLN has been demonstrated [44]. Hence, we believe that the modification in T cell subsets differentiation induced by the VSL#3 in the intestine of NOD mice was responsible for reduction of Teff/Treg cell ratio in the PLN and protection from T1D in those mice. Interestingly, the protective Teff/Treg cell balance was not found in the pancreatic islets infiltrates of VSL#3-treated NOD mice, thus suggesting that islet-reactive T cell number and pathogenicity are controlled within PLN from where a reduced number of islet-reactive Teff cells may migrate to the pancreatic tissues.

Overall, our results indicate that the intestinal mucosa of NOD mice is characterized by inflammasome activation and chronic inflammation possibly related to microbiota composition. VSL#3 treatment alone protects against T1D by altering microbiota composition, thus dampening intestinal inflammation and restoring gut immune homeostasis and the protective Teff/Treg cell balance in the gut mucosa and PLN. Those findings provide a proof of concept that modulation of the gut environment can control T1D pathogenesis by affecting intestinal immune cells. Moreover, our findings could pave the way for new therapeutic approaches aimed at preventing and/or treating human T1D by using probiotics to modulate intestinal immunity.

## Supplementary Material

The Supplementary Material contains two Figures that illustrate the gating strategies for identification of dendritic cell and T cell subsets in different organs (intestinal mucosa, pancreatic lymph nodes and pancreatic islets).
