# Supplementary material for: Oral Probiotic VSL#3 Prevents Autoimmune Diabetes by Modulating Microbiota and Promoting Indoleamine 2,3-Dioxygenase-Enriched Tolerogenic Intestinal Environment
Source: J Diabetes Res. 2015 Dec 8;2016:7569431. doi: 10.1155/2016/7569431 (PMC4686713; doi:10.1155/2016/7569431)
Supplement: Supplementary file 1 — The Supplementary Material contains two Figures that illustrate the gating strategies for identification of dendritic cell and T cell subsets in different organs (intestinal mucosa, pancreatic lymph nodes and pancreatic islets). [file 7569431.f1.pdf]

Supplementary Figure 1

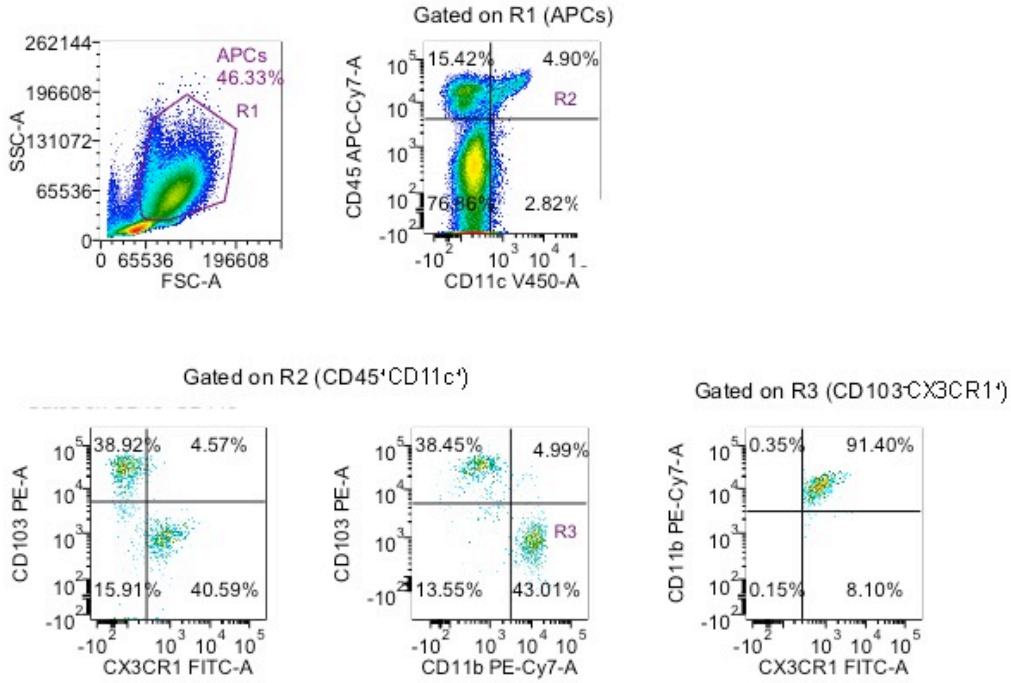

Figure S1: gating strategy for intestinal dendritic cell subsets. Mononuclear cells were isolated from the small and large intestines as previously described. After the removal of Peyer's patches, the intestines were flushed with PBS, opened longitudinally and predigested twice with 5 mM EDTA and 1 mM DTT for 20 min at 37°C. After removing epithelial cells and fat tissue, the intestines were cut into small pieces and incubated in HBSS containing 0.5 mg/ml Collagenase D (Roche), 1 mg/ml Dispase II (Roche) and 5 U/ml DNase I (Sigma) for 20 min at 37°C in a shaking incubator. The digested tissues were washed, resuspended in 5 ml of 40% Percoll (Sigma) and overlaid on 2.5 ml of 80% Percoll in a 15-ml Falcon tube. Percoll gradient separation was performed by centrifugation at 1000 g for 20 min at 20°C. The interface cells were collected and stained for FACS analysis with the following monoclonal antibodies: V450-conjugated hamster mAb anti-mouse CD11c, a PE-Cy7 conjugated rat mAb anti-mouse CD11b and a PE-conjugated rat mAb anti-mouse CD103 (all from BD Biosciences, San Josè, CA, USA). For CX3CR1 staining, we used a primary rabbit polyclonal antibody, anti-CX3CR1 (Abcam, Cambridge, UK), and a secondary FITC-conjugated F(ab')2 anti-rabbit IgG antibody (eBioscience, San Diego, CA, USA). The flow cytometry data were acquired using a FACScan and analyzed with CellQuest software (BD Biosciences, CA, USA).

Supplementary Figure 2

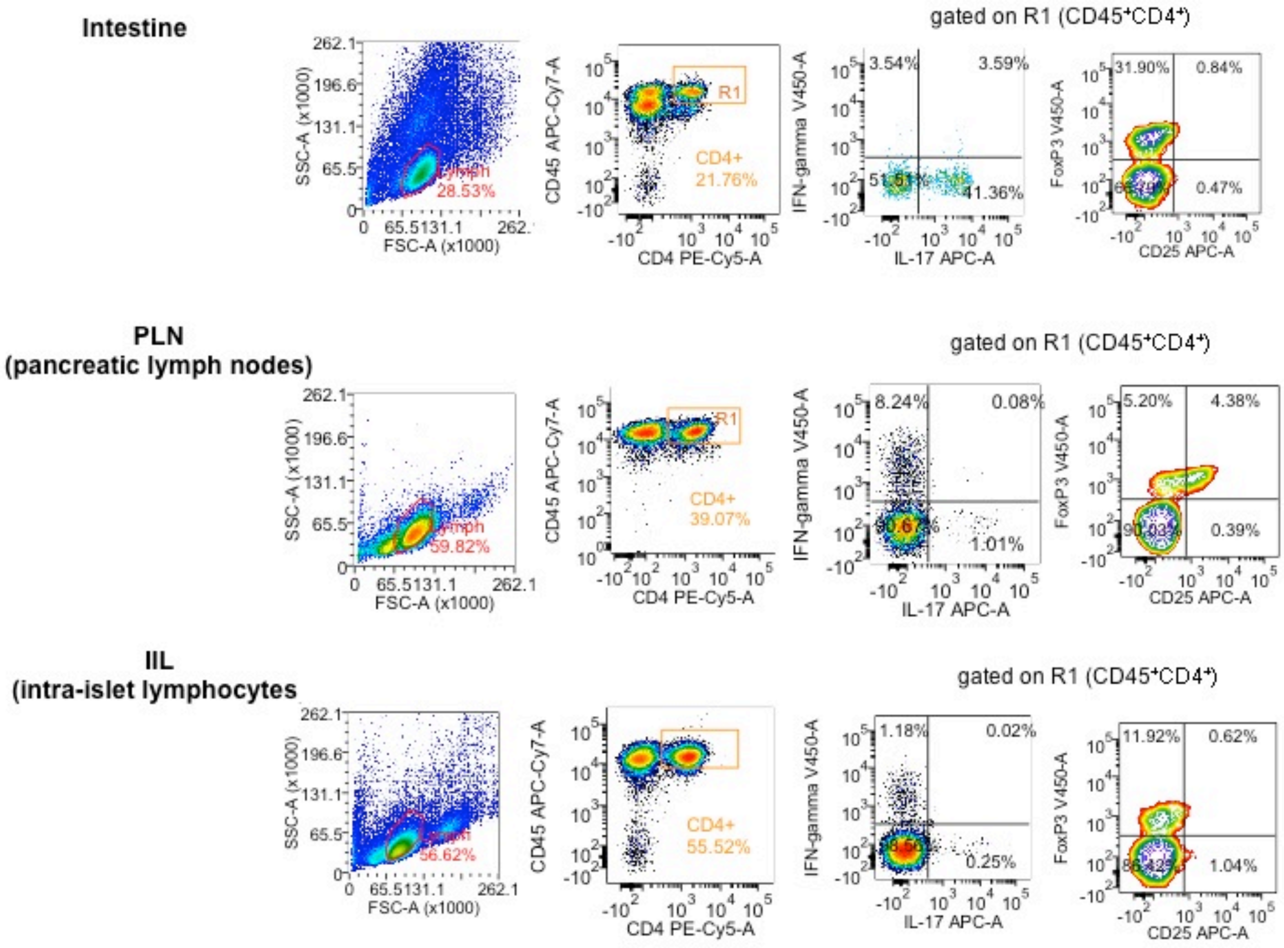

Figure S2: gating strategy for T cell subsets in the intestine, pancreatic lymph nodes (PLN) and intra-islet lymphocytes (IIL). Mononuclear cells were isolated from the small and large intestines as in FigureS1. Lymph node cells were isolated by mechanical disruption of the tissue. Pancreatic intra-islet lymphocytes were isolated by a 3-step digestion with 1 mg/ml Collagenase IV (Gibco) followed by extensive washing with HBSS containing 5% FBS. Single cell suspensions were stimulated with 50 ng/ml phorbol 12-myristate 13-acetate (PMA) and 1 μg/ml ionomycin (both from Sigma-Aldrich, St. Louis, MO, USA) for 4 hours in the presence of 10 μg/mL Brefeldin A (Sandoz, NJ, USA) followed by fixation and permeabilization with 2% Paraformaldehyde (PFA) and 0.5% Saponin (both from Sigma-Aldrich, St. Louis, MO, USA). After stimulation, the cells were stained with a PerCP-Cy5.5 conjugated anti-CD4 mAb, fixed and permeabilized (Cytofix/Cytoperm kit, BD Biosciences, San Josè, CA, USA) and stained with V450-conjugated anti-IFN-γ and APC-conjugated anti-IL17A mAbs. To detect the FOXP3 intracellular marker, cells were stained with PerCP-Cy5.5 anti-mouse CD4 and APC-conjugated anti-mouse CD25 mAbs against surface molecules and then fixed (Foxp3 Staining Buffer set, eBioscience, San Diego, CA, USA), permeabilized and stained with V450-conjugated anti-FOXP3 mAb. The flow cytometry data were acquired using a FACScan and analyzed with CellQuest software (BD Biosciences, CA, USA).
